# De novo transcriptome profiling and development of novel secondary metabolites based genic SSRs in medicinal plant *Phyllanthus emblica* L. (Aonla)

**DOI:** 10.1038/s41598-023-44317-x

**Published:** 2023-10-12

**Authors:** Bhuvnesh Kapoor, Megha Sharma, Rajnish Sharma, Ashwini Zadokar, Anamika Thakur, Parul Sharma, Suresh Kumar, K. Pung Rozar, Kewat Sanjay Kumar, Nagaraj Hegde, Devendra Pandey

**Affiliations:** 1 Department of Biotechnology, Dr YS Parmar University of Horticulture & Forestry, Nauni, Solan, HP 173 230 India; 2 University Institute of Biotechnology, Chandigarh University, Mohali, Punjab 140 413 India; 3 Department of Forestry, Mizoram University, Aizawl, Mizoram 796 004 India; 4 Department of Botany, University of Allahabad, Prayagraj, UP 211 002 India; 5 Division of Crop Improvement, ICAR-Central Institute for Subtropical Horticulture, Lucknow, UP 226 101 India

**Keywords:** Biotechnology, Plant sciences

## Abstract

*Phyllanthus emblica* (Aonla, Indian Gooseberry) is known to have various medicinal properties, but studies to understand its genetic structure are limited. Among the various secondary metabolites, ascorbic acid, flavonoids, terpenoids, phenols and tannins possess great potential for its pharmacological applications. Keeping this consideration, we assembled the transcriptome using the Illumina RNASeq500 platform, generating 39,933,248 high-quality paired-end reads assembled into 1,26,606 transcripts. A total of 87,771 unigenes were recovered after isoforms and unambiguous sequences deletion. Functional annotation of 43,377 coding sequences against the NCBI non-redundant (Nr) database search using BlastX yielded 38,692 sequences containing blast hits and found 4685 coding sequences to be unique. The transcript showed maximum similarity to *Hevea brasilensis* (16%), followed by to *Jatropha curcas* (12%). Considering key genes involved in the biosynthesis of flavonoids and various classes of terpenoid compounds, thirty EST-SSR primer sequences were designed based on transcriptomic data. Of which, 12 were found to be highly polymorphic with an average of 86.38%. The average value for marker index (MI), effective multiplicity ratio (EMR), resolution power (Rp) and polymorphic information content (PIC) was 7.20, 8.34, 8.64 and 0.80, respectively. Thus, from this study, we developed newly EST-SSRs linked to important genes involved in the secondary metabolites biosynthesis that will be serving as an invaluable genetic resource for crop improvement including the selection of elite genotypes in *P. emblica* and its closely related Phyllanthaceae species.

## Introduction

*Phyllanthus emblica* L. (syn. *Emblica officinalis* Gaertn.) also known as Indian gooseberry or aonla is a deciduous tree of the family Phyllanthaceae, distributed across the tropical and subtropical regions comprising over 800 species worldwide and over 50 species in India^1^. It is well known that *P. emblica* fruit is one of the richest natural sources of vitamin C^2^ and also contains several other vital bioactive phytoconstituents such as flavonoids; gallic acid, ellagic acid, rutin, quercetin and catechol^3^, terpenoids such as phyllaemblicins B, phyllaemblicins C, glochicoccinoside D^4^, tannins; mucic acid 1,4-lactone 3-*o*-gallate, isocorilagin, chebulanin, chebulagic acid and isomallotusinin^5^, respectively. *P. emblica* has been used as medicine and nutritional tonic in traditional medicine systems such as Ayurveda, Unani and Sidha to cure various infectious and non-infectious diseases^6^. Due to the presence of a variety of bioactive compounds in *P. emblica* extract, numerous therapeutic effects have been reported such as antimicrobial, antioxidant, anti-inflammatory, analgesic, antipyretic, adaptogenic, hepatoprotective, antitumor, antiulcerogenic and immunomodulatory activities^7–9^. From a pharmacological perspective, phenols and terpenoids are the major secondary metabolites of *P. emblica* while its high ascorbic acid content can be recommended as a high-quality and low-cost alternative for securing nutritional requirements.

Although *P. emblica* is well known to provide various medicinal benefits, only limited studies have been conducted to understand its genetic structure. To date, only 21 genomic^8, 10^ and 52 genic SSRs^11^ were reported in *P. emblica* that have been used for characterizing different populations. These microsatellite markers, however, are insufficient to investigate the genetic structure, variability and gene flow within the *P. emblica* population. Therefore, the development of molecular markers is essential for the characterization of *P. emblica* germplasm and phylogenetic studies of the species. This will help us better understand the genetics of *P. emblica* and enable the more effective use of a variety of germplasm for breeding programs.

Recent developments in sequencing technology offer enormous potential for genomic analysis and gene function study in both model and non-model organisms. Nowadays, de novo assembly is growing in popularity since it is a quick and cost-effective method for short reads when reference genomes are not available^12^. RNA-Seq platforms provided an opportunity to mine important molecular markers such as SNPs and SSRs at a much lower cost. RNA-Seq generates millions of short tags and subsequently assembled them, which can help to interpret genome and transcriptome sequences. The availability of transcriptome assembly and an adequate number of EST sequence data helps in the development of EST-SSR markers. Further, EST-SSRs are increasingly used for the evaluation of genetic relationships because they are abundant in gene-rich regions, co-dominant, highly polymorphic and easily transferable among phylogenetically related species^13^. Therefore, in this study, Illumina NextSeq500 sequencing technology was utilized to characterize the transcriptome of *P. emblica* shoot and to develop EST-SSR markers which can be used to evaluate the genetic diversity, linkage map construction and marker-assisted breeding. This study will provide valuable insights into the genetic structure of *P. emblica* that can be utilized in an improved breeding program.

## Materials and methods

### Plant material, RNA and DNA extraction

*Phyllanthus emblica* L. shoot tissues were procured from a fully grown healthy plant located at Mizoram University, Aizawl and were treated with 0.1% DEPC treated water followed by snap-frozen in liquid nitrogen and stored in a deep freezer (− 80 °C) until further use. For Illumina Sequencing, the total RNA was isolated from the shoot samples using the modified CTAB and lithium chloride (LiCl) method^14^. The concentration and purity of RNA samples were analyzed on 1% denaturing RNA agarose gel and bio-spectrophotometer (Eppendorf, Germany), respectively.

**Figure 1:**
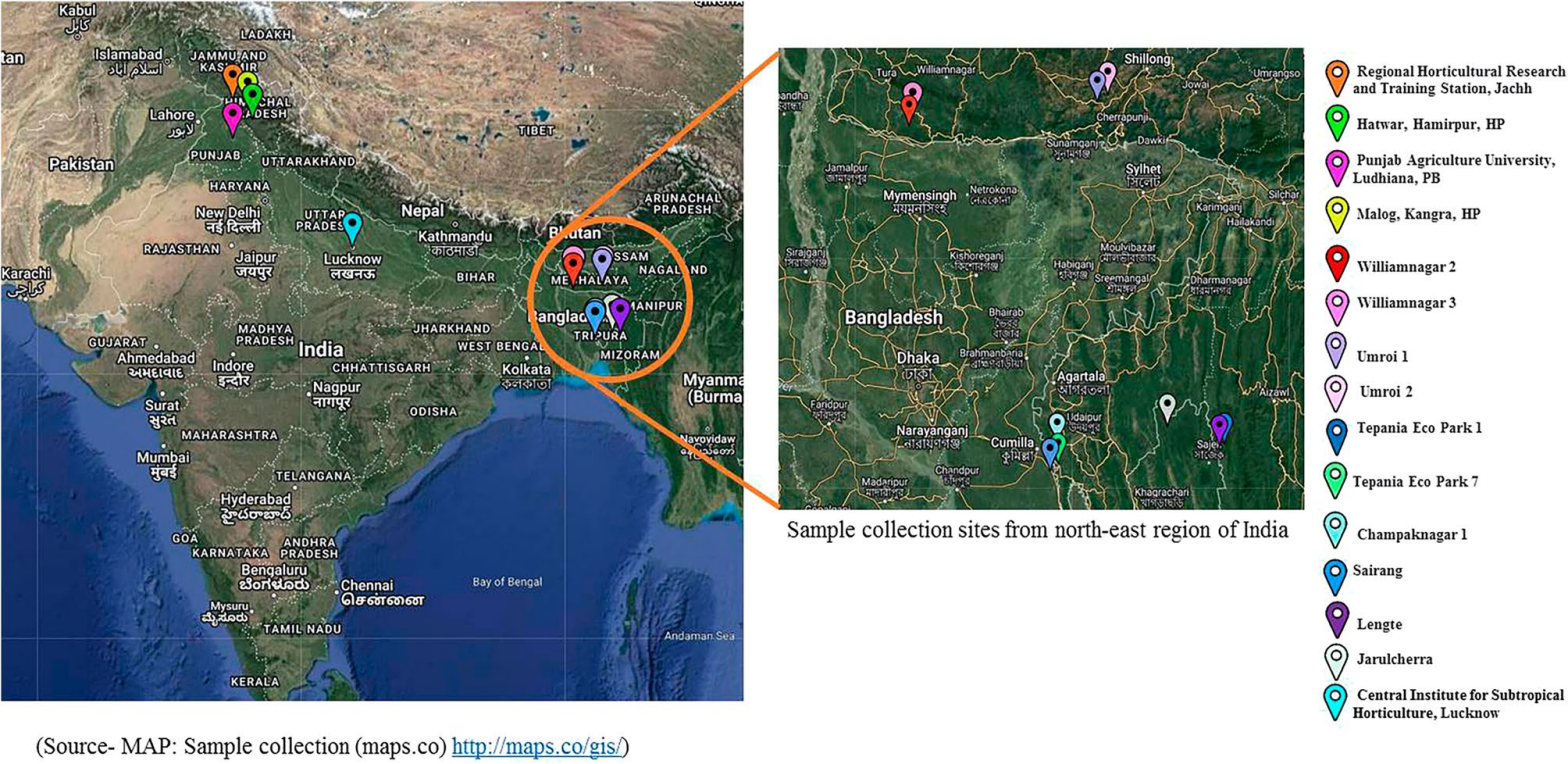
Map depicting different locations from where experimental material procured in the study.

To examine the polymorphism of EST-SSR markers, total of 30 leaf samples were collected from different locations comprising nine commercial varieties and twenty-one wild species of *Phyllanthus emblica* L. which has listed in Table 1 (Fig. 1) and stored at − 80 °C until DNA extraction. Two grams of leaves were ground in liquid nitrogen and genomic DNA was extracted using the CTAB method described by Doyle and Doyle^15^ with modification. The DNA was quantified with a bio-spectrophotometer (Eppendorf, Germany) and 0.8% agarose gel electrophoresis analysis.

**Table 1:** Details of *P. emblica* experimental material.

S. no.	Genotypes	Location	Coordinates	Elevation (m)	State
1.	NA6	Central Institute for Subtropical Horticulture, Lucknow	26° 79′ 79.461″ N	130	Uttar Pradesh
			80° 93′ 78.937″ E		
2.	NA7	Central Institute for Subtropical Horticulture, Lucknow	26° 79′ 79.461″ N	130	Uttar Pradesh
			80° 93′ 78.937″ E		
3.	NA10	Central Institute for Subtropical Horticulture, Lucknow	26° 79′ 79.461″ N	130	Uttar Pradesh
			80° 93′ 78.937″ E		
4.	L52	Central Institute for Subtropical Horticulture, Lucknow	26° 79′ 79.461″ N	130	Uttar Pradesh
			80° 93′ 78.973″ E		
5.	Chakaiya	Central Institute for Subtropical Horticulture, Lucknow	26° 79′ 79.461″ N	130	Uttar Pradesh
			80° 93′ 78.937″ E		
6.	Kanchan	Regional Horticultural Research and Training Station, Jachh	32° 28′ 08″ N	428	Himachal Pradesh
			75° 86′ 21″ E		
7.	HaathiJhool	Regional Horticultural Research and Training Station, Jachh	32° 28′ 08″ N	428	Himachal Pradesh
			75° 86′ 21″ E		
8.	Banarsi	Regional Horticultural Research and Training Station, Jachh	32° 28′ 08″ N	428	Himachal Pradesh
			75° 86′ 21″ E		
9.	Krishna	Regional Horticultural Research and Training Station, Jachh	32° 28′ 08″ N	428	Himachal Pradesh
			75° 86′ 21″ E		
10.	PUN1(PUN)	Punjab Agricultural University, Ludhiana	30° 90′ 09.71″ N	247	Punjab
			75° 85′ 72.698″ E		
11.	PUN2(PUN)	Punjab Agricultural University, Ludhiana	30° 90′ 09.71″ N	247	Punjab
			75° 85′ 72.698″ E		
12.	PUN3(PUN)	Punjab Agricultural University, Ludhiana	30° 90′ 09.71″ N	247	Punjab
			75° 85′ 72.698″ E		
13.	HPK2(HP)	Malog, Kangra	32° 01′ 03″ N	807	Himachal Pradesh
			76° 47′ 22″ E		
14.	HPK3(HP)	Malog, Kangra	32° 01′ 03″ N	807	Himachal Pradesh
			76° 47′ 22″ E		
15.	HPK4(HP)	Malog, Kangra	32° 01′ 03″ N	807	Himachal Pradesh
			76° 47′ 22″ E		
16.	HPH3(HP)	Hatwar, Hamirpur	31° 57′ 31″ N	798	Himachal Pradesh
			76° 69′ 76″ E		
17.	HPH4(HP)	Hatwar, Hamirpur	31° 54′ 39″ N	798	Himachal Pradesh
			76° 47′ 91″ E		
18.	HPH5(HP)	Hatwar, Hamirpur	31° 54′ 39″ N	798	Himachal Pradesh
			76° 47′ 91″ E		
19.	TPN1	Tepania Eco Park	23° 33′ 32.51″ N	47	Tripura
			091° 26′ 57.09″ E		
20.	TPN7	Tepania Eco Park	23° 33′ 20.96″ N	53	Tripura
			091° 27′ 00.81″ E		
21.	JRL1	Jarulcherra	23° 58′ 41.72″ N	98	Tripura
			092° 02′ 11.78″ E		
22.	CPG1	Champaknagar	23° 48′ 26.69′′N	79	Tripura
			091° 30′ 51.44″ E		
23.	CPG5	Champaknagar	23° 48′ 26.31″ N	96	Tripura
			091° 31′ 04.86″ E		
24.	JRL2	Jarulcherra	23° 58′ 30.65″ N	100	Tripura
			092° 02′ 27.75″ E		
25.	UMR 1	Umroi	25° 42′ 40.92′′N	880	Meghalaya
			91° 59′ 26.86″ E		
26.	UMR 2	Umroi	25° 39′ 16.68″ N	1094	Meghalaya
			091° 57′ 32.98″ E		
27.	WNG 2	Williamnagar	N25° 30′ 29.64′′ N	269	Meghalaya
			E90° 37′ 31.25′′ E		
28.	WNG 3	Williamnagar	N25° 30′ 30.57′′ N	270	Meghalaya
			E90° 37′ 31.63′′ E		
29.	SAIRANG 1	Sairang	23° 48′ 26.19′′ N	189	Mizoram
			92° 39′ 24.27′′ E		
30.	LENGTE	Lengte	23° 47′ 40.1′′ N	309	Mizoram
			092° 36′ 12.4″ E

### RNA sequence library preparation and sequencing

The RNA-Seq paired-end sequencing library was prepared from the purified RNA samples after pooling them in equimolar concentration^16^ using TruSeq standard mRNA sample prep kit (Illumina, California, USA). Briefly, mRNA was enriched from the total RNA using poly-T attached magnetic beads, followed by enzymatic fragmentation, 1st strand cDNA conversion using SuperScript II and Act-D mix to facilitate RNA-dependent synthesis. The 1st strand cDNA was then synthesized to the second strand using a second strand mix. The dscDNA was then purified using AMPure XP beads followed by A-tailing, adapter ligation and then enriched by a limited number of PCR cycles. The PCR enriched library was analyzed on a 4200 Tape station system (Agilent Technologies, Chandigarh, India) using sensitivity D1000 screen tape as per manufacturer instructions. The prepared library was sequenced on the Illumina NextSeq500 platform to produce 150 bp paired-end reads.

### De novo transcriptome assembly and data clustering

The sequence raw data were processed to obtain high-quality concordant reads using Trimmomatic v0.38^17^ and in-house script to remove the adapter, ambiguous reads (reads with unknown nucleotides “N” larger than 5%) and low-quality sequences (reads with more than 10% quality threshold (QV) < Phred score). Further, these high-quality reads (QV ≥ 20) were assembled into transcripts using Trinity de novo assembler (v2.8.4)^18^ with kmer of value 25. The assembled transcripts were then clustered together using CD-HIT-EST 4.6^19^ to remove the isoform produced during assembly. The resulting sequences were identified as unigenes and were considered for downstream analysis.

### Sequence annotation and gene ontology (GO) analysis

TransDecoder-v5.3.0 (http://transdecoder.github.io/) was used to predict coding sequences (CDS) from the retrieved unigenes and identified candidate coding regions within unigene sequences. The functional annotation of coding sequences was performed using the DIAMOND program^20^ which is a BLAST-compatible local aligner for mapping translated DNA query sequences and finds homologous sequences for transcripts against non-redundant protein database from NCBI. GO analysis of identified coding sequences was carried out using the Blast2GO program. GO mapping was executed to retrieve GO categories for functionally annotated transcripts. BlastX result accession IDs were used to retrieve gene name symbols, identified gene names or symbols are then explored in the species-specific appearances of the gene product tables of the GO database. BlastX result accession IDs are used to retrieve UniProt IDs utilizing PIR that incorporates PSD, UniProt, SwissProt, TrEMBL, RefSeq, PDB and GenPept databases.

### Development and detection of EST-SSR markers

The Microsatellite searching tool (MISA, http://pgrc.ipk-gatersleben.de/misa) was used for the identification of potential microsatellites from all the unigenes found in the transcriptome sequence of *P. emblica.* Primer 3 software (http://primer3.sourceforge.net/releases.php) was used to design the EST-SSR markers by optimizing the primer parameters such as; primer length range between 18 and 23 bp, GC content 40 ± 60%, product size range between 100 and 300 bp and annealing temperature ranging from 55 to 66 °C. A total of 30 primer pairs were randomly selected, designed and used to study the germplasm characterization of *P. emblica.*

### PCR amplification and PAGE analysis

A total of 30 EST-SSR markers were designed to evaluate the genetic diversity among the studied aonla genotypes. For PCR analysis, a final volume of 10 µl reaction mixture containing 1X Taq polymerase buffer with MgCl_2_, 0.2 mM dNTP, 0.3U Taq DNA polymerase, 10 pmol SSR primers (Eurofins, India), and 50 ng/l of genomic DNA was utilized. The following thermal profile was used for the PCR amplification in the thermal cycler (Applied Biosystems, USA): initial denaturation at 94 °C for 5 min; 35 cycles of 94 °C for 1 min; annealing at varying temperatures depending on the primer pair for 45 s; and extension at 72 °C for 45 s; followed by final extension of products at 72 °C for 8 min. Following polyacrylamide gel analysis of the PCR results and assessment of amplicon size were measured against a 50 bp DNA ladder (GeNei, Bangalore, India).

### Genetic diversity analysis

To assess the genetic diversity among 30 genotypes of aonla germplasm (Supplementary Table 1), the dendrogram was constructed based on the unweighted pair group method of the arithmetic mean (UPGMA) by using Jaccard’s similarity coefficient with the help of DARwin software ver.6. Further, factorial analysis and a Neighbour-Joining tree were constructed with the help of DARwin software ver.6^21^. Program POPGENE 1.32 was used to determine each primer’s polymorphic information content (PIC), Marker Index (MI), effective multiplex ratio (EMR), resolving power (Rp), estimates of gene diversity for each population across all loci in terms of alleles per locus (Na), the effective number of alleles (Ne), Shannon’s information index (I), observed heterozygosity (Ho) and expected heterozygosity (He)^22, 23^.

### Research involving plants

The necessary permissions for procuring the *Phyllanthus emblica* germplasm used in the current study have taken from the mentioned collection sites. The said material comprising of commercial genotypes is authenticated and validated by Rajnish Sharma while the wild genotypes are being authenticated, validated and maintained by Suresh Kumar at Mizoram University. All experimental research and field studies on plants (either cultivated or wild), including the collection of plant material were carried out in accordance with relevant institutional, national, and international guidelines and legislation.

## Results and discussion

### De novo transcriptomic assembly

The transcriptome sequencing of *P. emblica* using the Illumina NextSeq500 platform produced 39,933,248 Pair End (PE) reads, 5,991,900,473 bases and ~ 6 Gb total data. After trimming of adapter and removal of ambiguous sequences, high-quality reads were assembled into a total of 1,26,606 transcripts with a mean transcript length of 951 bp (Table 2). The de novo transcriptome assembly was best developed at k-mer 25 at 1418 bp N50 value. The maximum transcript length was found 7539 bp with a minimum transcript length of 201 bp (Table 2). After the removal of isoforms from the 1,26,606 transcripts, a total of 87,771 unigene sequences were retrieved. The average unigene length was found 884 bp with a maximum unigene length of 7539 bp (Table 2). From the first transcriptomic study of leaf and flower tissue of *P. emblica* using Illumina Hiseq2000 platform 1,34,205 unigene sequences and 89,242 singletons with an average contig length of 278 bp reported were reported by Kumar et al.^24^. While, in another leaf transcriptome study using Illumina Hiseq4000, a total of 76,881 non-redundant genes were reported by Liu et al.^11^.

**Table 2:** Summary of transcriptome data generated in Illumina NextSeq500 for *P. Emblica* L.

Sr. no.	Description	Transcript	Unigene	CDS
1	Number of total transcripts	1,26,606	87,771	43,377
2	Total transcript length (bp)	12,03,789,344	77,595,170	36,316,356
3	N50	1418	1364	1029
4	Maximum length (bp)	7539	7539	6300
5	Minimum length (bp)	201	201	261
6	Average length (bp)	951	884	837

*CDS* coding sequences, *bp* base pairs.

### Prediction of coding sequences and function annotation

TransDecoder (v5.3.0) was used to find the coding sequences within a total of 87,771 Unigenes. As a result, a total number of 43,377 coding sequences with a maximum coding sequence length of 6300 bp with an average length of 837 bp were obtained (Table 2). After finding the coding regions based on various structural and positional parameters, it became important to find the functional information associated with assembled coding regions. Hence, coding regions were aligned by DIAMOND (BlastX alignment tool) to find the homologous sequences against NR (Non-Redundant) protein database from NCBI. Out of 43,377 transcripts, 38,692 coding sequences found Blast hit and 4685 were found unique. Reciprocally, Kumar et al.^24^ have shown a similarity of 47,276 sequences with the NCBI-NR protein database. In terms of similarity of coding sequences among other species, maximum of 16% of transcripts showed similarity with *Hevea brasiliensis* followed by *Jatropha curcas* (12%), *Manihot esculenta* (10%), *Ricinus communis* (9%) *Populus trichocarpa* (9%), *Populus eupharatica* (5%), *Citrus sinesis* (2%), *Theobroma cacao* (2%), *Vitis vinifera* (1%) and *Quercus suber* (1%) (Supplementary Fig. 1). In contrast, Kumar et al.^24^ reported the maximum transcripts showed homology with genes of *Vitis vinifera* (29%) followed by *Oryza sativa* (14.35%). The resulting similarity of *P. emblica* coding sequences with four Euphorbiaceae plants viz. *Hevea brasiliensis, Jatropha curcas, Manihot esculenta* and *Ricinus communis* reveal the similarities in gene architecture among Phyllanthaceae and Euphorbiaceae families.

### Gene ontology analysis

GO assignments were used to classify functions of predicted coding sequences and also provided ontology of defined categories representing protein properties which are grouped into three main domains: Biological Process (BP), Molecular Function (MF) and Cellular Components (CC). A total of 11,134 coding sequences were annotated using Blast2GO analysis. Molecular Functions (MF) was found to have the highest number of 8727 coding sequences associated with it followed by 7596 coding sequences in Biological Processes (BP). While the least number of coding sequences were found associated with cellular components (6135). In concordance, Kumar et al.^24^ also reported that maximum predicted unigenes were found associated with molecular functions followed by cellular functions and biological processes respectively, following significant hits of unigenes against the NR database. In the category of MFs of GO, organic cyclic compound binding (3296/16%), heterocyclic compound binding (3292/16%), ion binding (2959/14%), transferase activity (2157/10%), small molecule binding (1908/9%), hydrolase activity (1796/9%), carbohydrate derivative binding (1585, 8%), catalytic activity on proteins (1439/7%), drug binding (1343/6%) and oxidoreductase activity (1067/5%) were annotated GO categories. Under the biological process GO category, organic substance metabolic process (4628/20%), cellular metabolic process (4407/19%), primary metabolic process (4381/19%), nitrogen compound metabolic process (3829/16%), biosynthetic process (2001/9%), the establishment of localization (1227/5%), oxidation–reduction process (1078/5%), regulation of cellular process (1019/4%), small molecule metabolic process (850/4%) were found most enriched categories. In the GO category of cellular components, membrane (3322/25%), organelles (2718, 21%), intracellular organelle (2666/20%), an intrinsic component of membrane (2531/19%), cytoplasm (1934/15%) were found most represented categories (Supplementary Fig. 2).

### Flavonoid and terpenoid biosynthesis

Phenylpropanoid pathway begins deamination of aromatic amino acid phenylalanine and leads to a synthesis of a wide range of phenolic acids as secondary metabolites while chalcone synthase generates the intermediated flavonoid compound called naringenin, further oxidation and hydroxylation of naringenin generates eriodictyol and dihydrotricetin respectively. Further enzyme catalytic reactions convert this product either into anthocyanidins or catechins. Catechins are flavonoids distributed in a variety of foods and herbs including tea, apples, persimmons, cacaos, grapes, and berries^25^. Since they have tremendous beneficial health implications for humans and also play an important part in plant growth and development. For full utilization of these compounds in food, medicine, and other purposes requires a thorough understanding of genes and distinct biosynthetic pathways of their production in cellular systems and this makes them a versatile target for metabolic engineering^26^. The study conducted by Zhang et al.^27^ listed the presence of flavones from the leaves and branches of *P. emblica* by isolating two acylatedflavanone glycosides [S-eriodictyol 7-*O*-(6″-*O-trans-p*-coumaroyl)-β-d-glucopyranoside and S-eriodictyol 7-*O*-(*6″-O-galloyl*)-β-D-glucopyranoside] together with a new phenolic glycoside, 2-(2-methylbutyryl) phloroglucinol 1-*O*-(6″-*O*-β-D-apiofuranosyl)-β-D-glucopyranoside. This study noted the existence of significant flavones in *P. emblica* leaves and branches, highlighting the need for further investigation in the crop’s transcriptomics and metabolomics areas. Aonla shoot tissue used in the current study's transcriptome analysis revealed 62 genes associated with the production of flavonoids (Supplementary Table 2).

Terpenoids also known as isoprenoids are secondary metabolites synthesized in plants through two non-homologous biosynthetic pathways; first, the cytosolic mevalonate pathway (MVA) leading to the synthesis of sesquiterpenoids (C15) and the second non-mevalonate or plastid methylerythritol 4-phosphate (MEP) pathway resulting in a synthesis of monoterpenoids (C10) and diterpenoids^28^. Transcriptome analysis revealed a total of 48 predicted unigenes involved in terpenoid biosynthesis. Among them, 27 unigenes were found associated with terpenoid backbone biosynthesis, 9 unigenes for sesquiterpenes, and 7 unigenes for diterpenoids followed by 5 unigenes for monoterpenoid biosynthesis. By taking into consideration of terpenoids’ chemical diversity and their health implication into account there is a great need to understand the molecular mechanisms involved in triterpenoid saponin production in planta to assist their exogenous engineering, pinpoint biosynthetic genes, transporters and transcription factors.

### Development and characterization of EST-SSR markers

**Figure 2:**
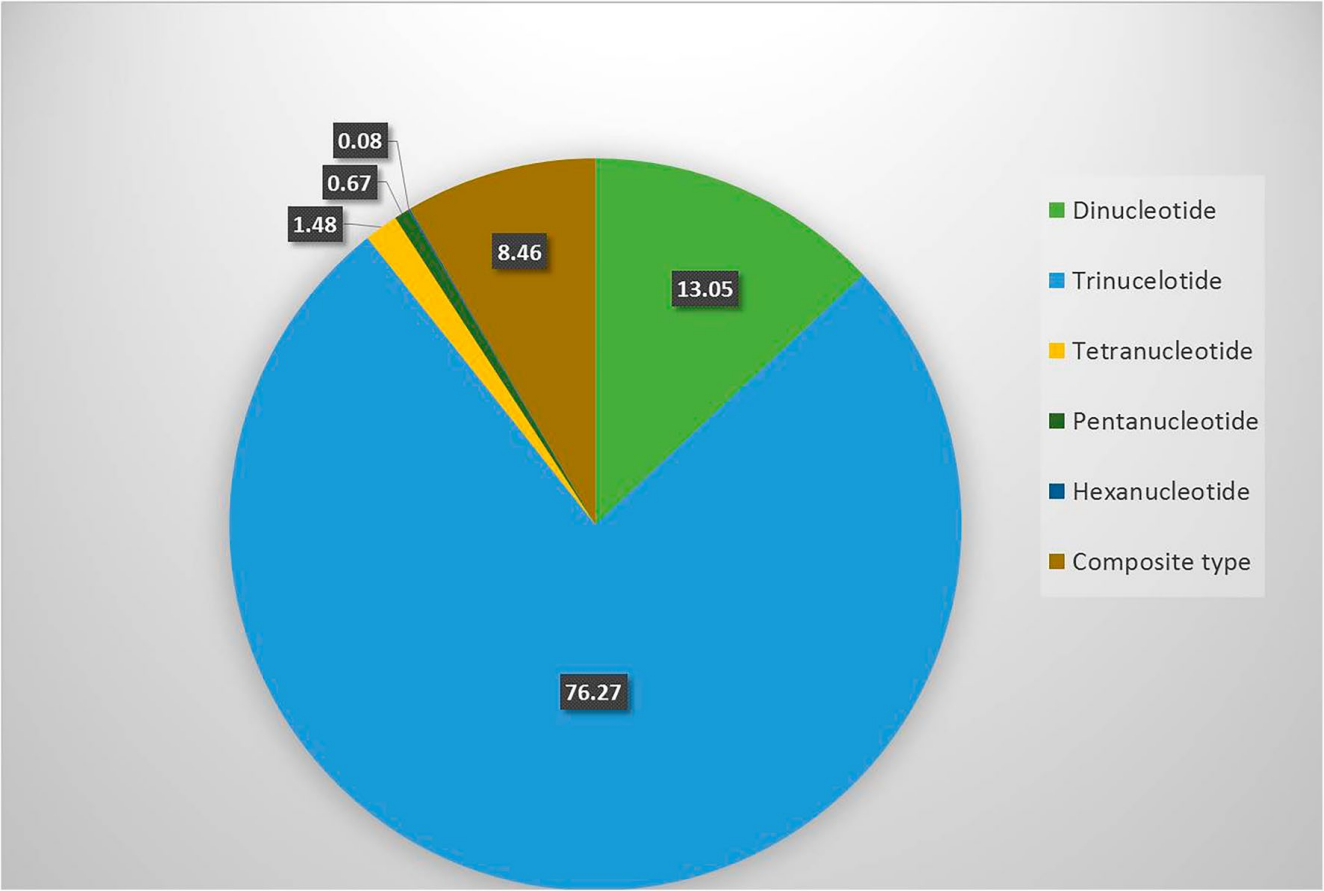
Frequency distribution of identified SSR motifs in the *P. emblica* transcriptome.

A total of 7477 SSR sequences were retrieved from the 87,771 unigenes of *P. emblica* and only 448 sequences with more than one SSR were reported. The frequency distribution of SSRs is mainly comprised of more than one repeat motif viz., tri-nucleotide repeats (76%), followed by di-nucleotide (13%), composite type (8.46%), tetra-nucleotide (1.48%), penta-nucleotide (0.67%) and hexa-nucleotide (0.08%) repeats, respectively (Fig. 2, Supplementary Table 3). Consequently, 30 of them were randomly selected for primer design using Primer3 software^28^.

22 of the 30 EST-SSR primer pairs showed successful amplification with genomic DNA of 30 *P. emblica* accessions with the desired product size while the remaining 8 primer pairs did not show any amplification. Out of 22 EST-SSRs, only 12 (40%) were found to be polymorphic and further used to assess the genetic diversity among the studied *P. emblica* genotypes (Supplementary Figs. 3, 4). These 12 EST-SSRs produced a total of 122 bands, of which 12 (9.83%) were monomorphic while 110 (90.16%) were polymorphic. The allele number for each primer ranged from 2 (PES-28) to 23 (PES-21), with an average of 10.17 and amplicon size varied from 100 to 480 bp (Table 3). However, Liu et al.^11^ reported that the average number of alleles per locus varied from 11 to 44 and the size of amplified product ranging from 104 to 297 bp while assessing the three populations of *P. emblica* using EST-SSR primers. Likewise, Pandey and Changtragoon^10^ and Geethika et al.^8^ studied and found that the number of alleles per locus varied from 4 to 7 and 2 to 9 with product size ranging from 150–236 bp to 130–330 bp, respectively while evaluating the genetic diversity among the two natural populations and 20 accessions of *P. emblica,* respectively. The variability in the allele number generated by primes may depend on the compatibility of the primer’s association with the plant genome as well as the components of each of the nitrogenous bases.

**Table 3:** Diversity statistics inferred in aonla (*P. emblica*) germplasm collected from different locations using EST-SSRs based on shoot transcriptome data.

Primer	Sequence	Ta (°C)	Allele size range (bp)	Total alleles	Allele frequency	No. of amplified bands			Poly%	PIC	EMR	Rp	MI	Na	Ne	I	Ho	He
						Total polymorphic monomorphic												
PES-21	F: TGAAACCACACCTCCACTTG R: TGGTGGTGATGGCAATAGAA	56	100–330	232	7.73	23	20	3	86.96	0.93	17.39	15.47	16.09	5.40	3.89	1.43	0.96	0.86
PES-25	F-GGCAAAGTTGGGACTGAAAA R-CCAAACCCAAACAAAACACC	53	170–195	78	2.60	5	4	1	80	0.74	3.20	5.20	2.36	2.40	1.96	0.69	0.72	0.58
PES-28	F-ACTCGTTGTCGGTCCATTTC R-TGCGAGCTCAGTAATTGTGG	57	140–240	40	1.33	2	1	1	50	0.50	0.50	2.67	0.25	2.80	2.50	0.94	0.65	0.73
PES-30	F-ATACGCGGAAAAGGTGACAG R-CAGATCCGTCCTTGGAGAAG	60	100–480	136	4.53	12	11	1	91.67	0.88	10.8	9.07	8.84	4	3.04	1.12	0.88	0.75
PES-32	F-CACATGGCACTGGAGCTAGA R-GCAAGGTGACTTCAGCAACA	58	150–220	144	4.80	10	9	1	90	0.85	8.10	9.60	6.85	2.20	2.08	0.74	1	0.65
PES-33	F-GAACATAAAGGCCAGGTGGA R-AATGCACACAAAAGGGAACC	54	180–295	200	6.67	13	12	1	92.31	0.89	11.08	13.33	9.87	3.40	2.80	1.05	0.98	0.75
PES-35	F-TTCCTCTCCACTTTCGGCTA R-AACGAAGGAGAAGCGATCAA	54	165–195	61	2.03	4	3	1	75	0.57	2.25	4.07	1.29	2.40	2.08	0.77	0.86	0.64
PES-40	F-TCAAGTAGCCACCCCAAAAC R-GTGGGACCCATATTCCTCCT	58	280–350	100	3.33	6	6	0	100	0.81	6	6.67	4.88	4.40	3.36	1.26	0.92	0.83
PES-42	F-ATGCCGTATCTTCACCGAAC R-CTTCAGGTTGTCAGCATCCA	57	250–380	75	2.50	10	10	0	100	0.83	10	5	8.29	3	2.47	0.93	0.73	0.68
PES-44	F-GTCTGTTTCGGTGGAGGAGA R-TATCGTCATTTGCCCAACAA	54	125–328	175	5.83	9	7	2	77.78	0.87	5.44	11.67	4.72	3.80	2.79	1.10	1	0.76
PES-48	F-GAACCAAAGCTGTCCCAGAA R-AGGGTCGTCAAAGAAGAGCA	54	137–220	127	4.23	14	13	1	92.86	0.87	12.07	8.47	10.44	3.60	2.76	1.07	0.88	0.76
PES-49	F-CCGTTGATTCGAAGGAGAAA R-AACTGCCTGCACACACACTC	55	140–235	187	6.23	14	14	0	100	0.89	14	12.47	12.51	3.60	3.29	1.14	0.94	0.79
Total				1555	51.83	122	110	12	1036.57	9.62	100.12	103.67	86.39	41	33.02	12.24	10.52	8.78
Average				129.58	4.32	10.17	9.17	1.00	86.38	0.80	8.34	8.64	7.20	8.20	6.60	2.45	2.10	1.76

*PES Phyllanthusemblica* shoot, *Ta* annealing temperature, *bp* base pairs, *Poly%* polymorphic percentage, *PIC* polymorphic information contents, *EMR* efective multiplex ratio, *Rp* resolving power, *MI* marker index, *Na* number of alleles, *Ne* effective number of alleles, *I* Shannon index, *Ho* observed heterozygosity and *He* expected heterozygosity.

The Polymorphism Information Content (PIC) value is used to determine the informativeness of a molecular marker; the higher the PIC value the more informative the primer. In the present study, the PIC values ranged from 0.50 (PES-28) to 0.93 (PES-21), with an average of 0.80. As evident from Table 3 the PIC value for each primer is equal to or greater than 0.5, indicating that all the primers are very informative and can be further utilized for germplasm characterization of *P. emblica*. Moreover, the highest percent polymorphism (100%) was recorded among three EST-SSR primers namely PES-40, PES-42 and PES-49, while the lowest percent polymorphism was observed in only one primer named PES-28 (50%) with an average of 86.38 percent polymorphism (Table 3). Additionally, the resolving power/discriminatory power (Rp), Marker Index (MI), and effective multiplex ratio (EMR) was also calculated for each polymorphic primer. The resolving power of a primer indicates the discriminatory potential of the primer to distinguish the genotypes or individuals. The resolving power of each primer ranged from 2.67 (PES 28) to 15.47 (PES 21), with an average of 8.64 (Table 3). Likewise, the effective multiplex ratio (EMR) was also calculated for all the 12 polymorphic primers and varied from 0.50 (PES 28) to 17.39 (PES-21), with an average of 8.34 (Table 3). Marker index (MI) is a feature of marker which explains the discriminatory power of a marker and is the product of PIC and EMR value. The maximum MI was recorded for primer PES-21 (16.09) and minimum for primer PES-28 (0.25) (Table 3).

The average number of alleles (Na), effective number of alleles (Ne), Shannon index (I), expected heterozygosity (He) and observed heterozygosity (Ho) was also estimated in the present study. The highest average number of alleles (Na), effective number of alleles (Ne), Shannon index (I) i.e. 5.40, 3.89 and 1.43, respectively were observed for the primer PES-21 while the lowest average number of alleles (Na) was recorded for primer PES-32 (2.20), the effective number of alleles (Ne) and Shannon index (I) for primer PES-25 i.e. 1.96 and 0.69, respectively (Table 3). The observed and expected heterozygosity for currently studied *P. emblica* genotypes was ranged from 0.65 to 1.00 and 0.58 to 0.86, respectively, with an overall average of 2.10 and 1.76, respectively. Similarly, in the previous studies, high level of genetic diversity at species level in terms of observed and expected heterozygosity was also estimated while characterizing the germplasm of *P. emblica* for example, Pandey and Changtragoon^9^ reported the Ho and He ranged from 0.360 to 0.760 and 0.499 to 0.806, respectively, by using six microsatellite markers in two natural population of *P. emblica.* Likewise, Geethika et al.^8^ and Liu et al.^11^ also recorded the Ho (0 to 1.00; 0.24 to 0.86) and He (0.401 to 0.825; 0.75 to 0.93) while assessing the genetic diversity of twenty and ninety *P. emblica* accessions using fifteen and twenty-one microsatellite markers, respectively.

### Genetic diversity analysis

**Figure 3:**
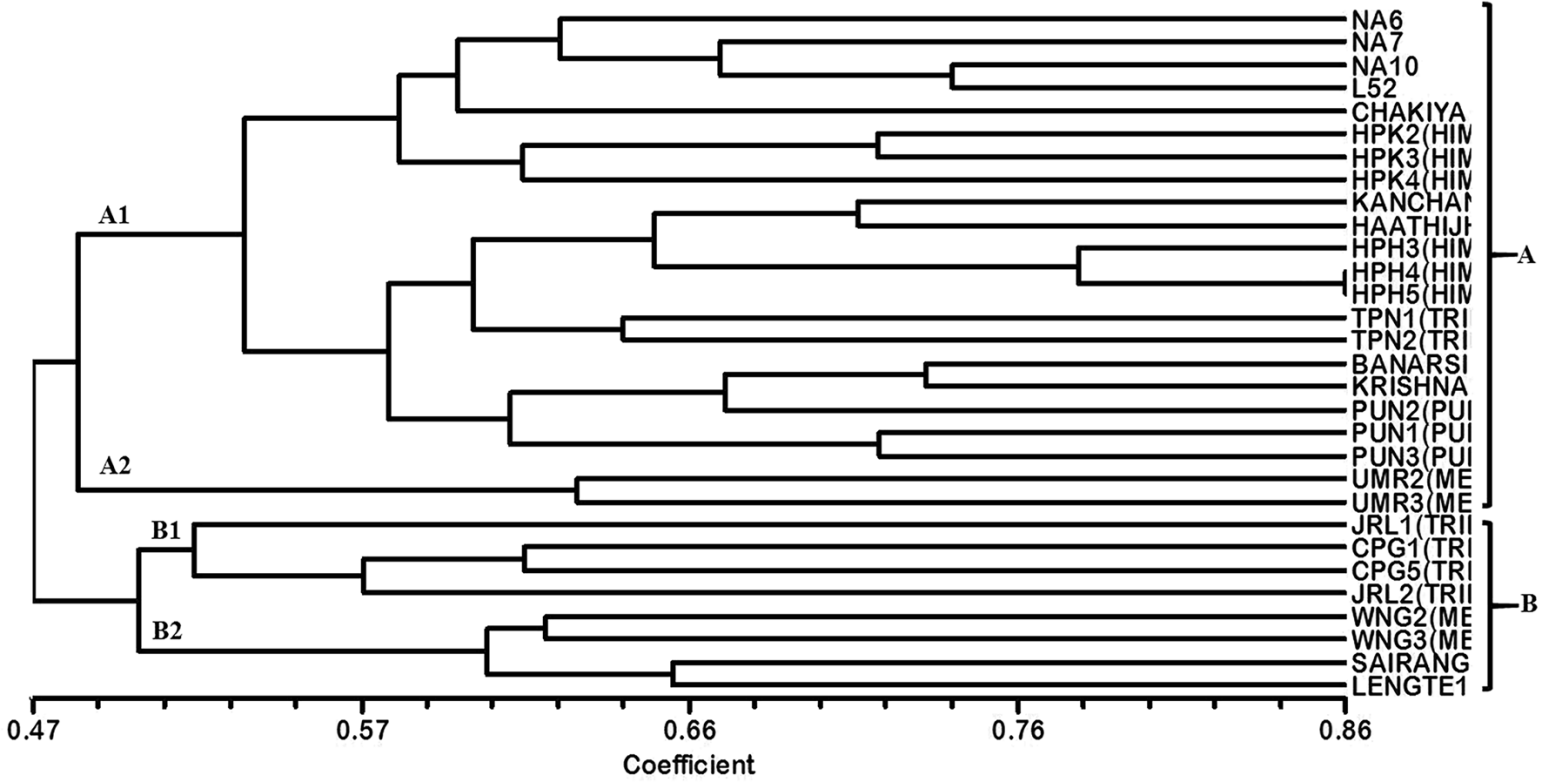
Dendrogram illustrating hierarchical clustering of aonla genotypes.

**Figure 4:**
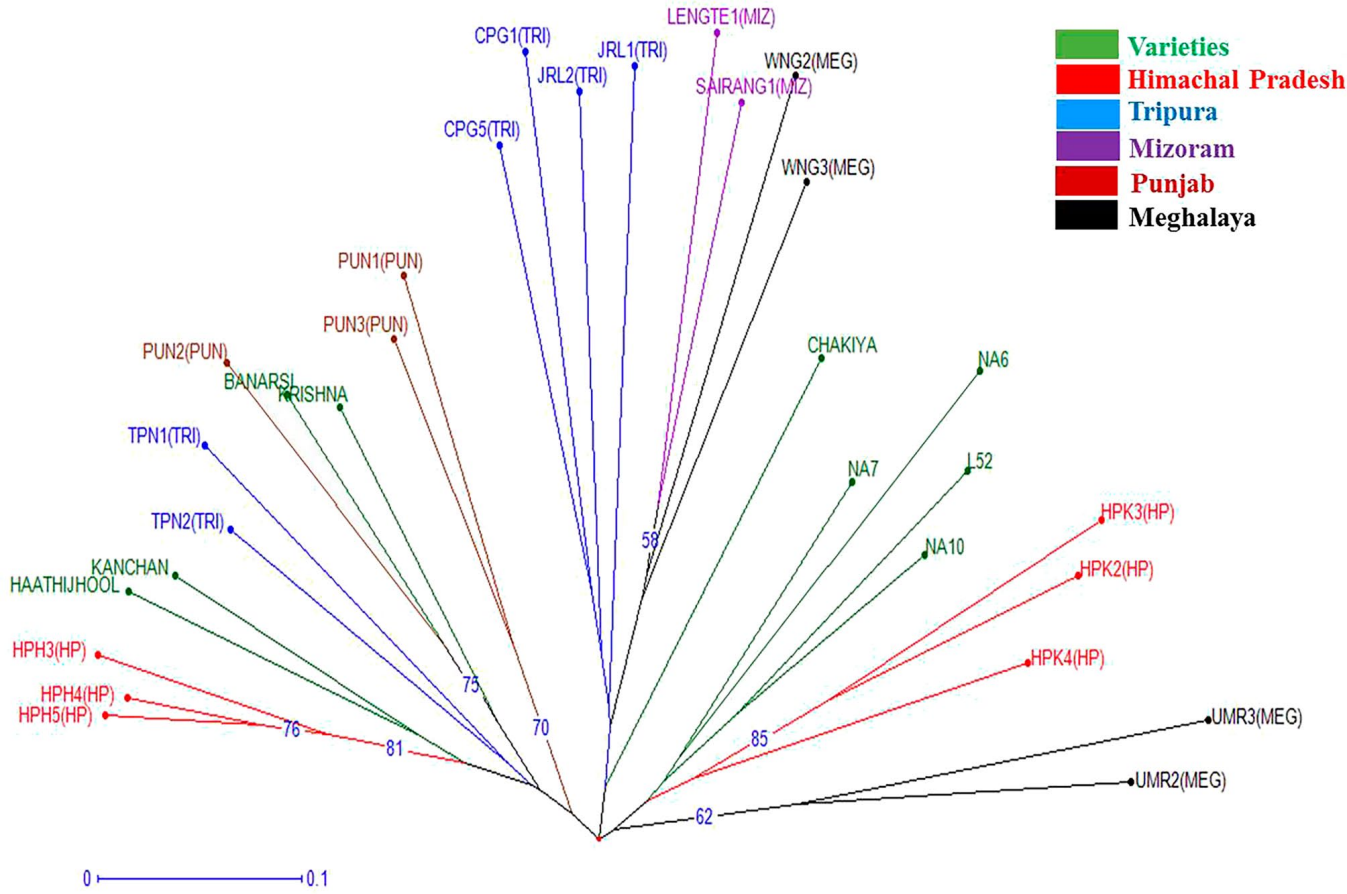
Neighbour-Joining phylogenetic tree of 30 aonla genotypes.

**Figure 5:**
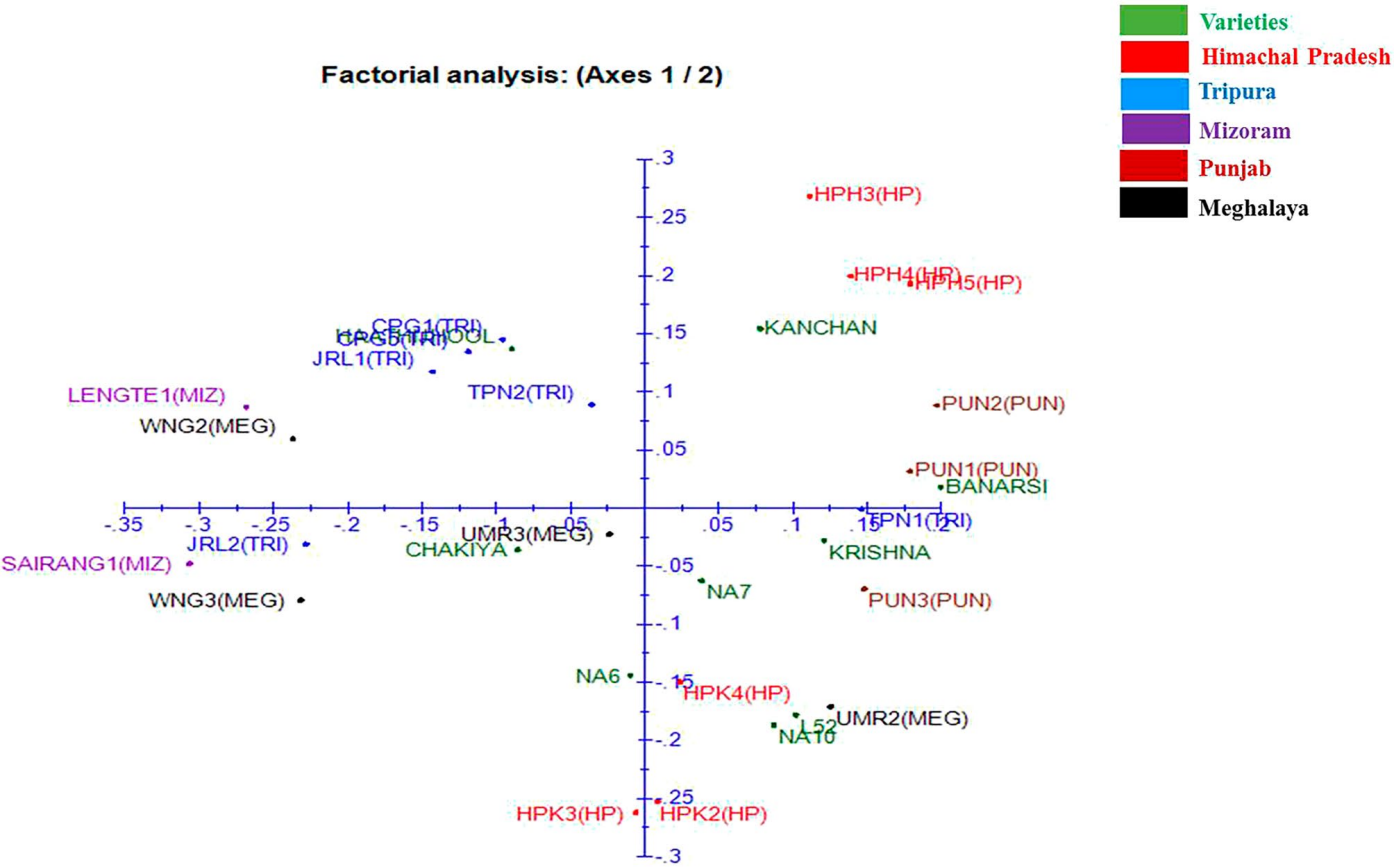
Factorial analysis of aonla genotypes.

Based on the data obtained using 12 informative EST-SSR primers, a dendrogram was generated (Fig. 3). The dendrogram is a diagrammatical representation in the form of a tree illustrating the arrangement of clusters generated by EST-SSR analysis using NTSYS software divided the genotypes under studies into two main clusters namely, A and B at a similarity coefficient of 0.47 as presented in Fig. 3. The major cluster A comprises of 22 aonla genotypes which further bifurcated at a similarity coefficient ~ 0.487 into two sub-groups A_1_ and A_2_. These results indicated the consistency of genetic structure with geographical distribution while showing occurrence of similar genetic variations within two individual groups as reported by Liu et al.^29^ showing 20 EST-SSR primers to group the 260 *P. emblica* accessions into two major clusters. The sub-group A_1_ consists of 20 genotypes which include all the commercial varieties and genotypes from Himachal, Punjab and Tripura. However, HPH4 and HPH5 from Himachal showed the highest similarity coefficient value of 0.96 indicating the presence of similar genetic base. Whereas, the sub-group A_2_ comprised only two genotypes i.e. UMR2 and UMR3 from Meghalaya. On the other hand, Cluster B consists of 8 *P. emblica* genotypes and is grouped into two sub-clusters namely, B_1_ and B_2_ at a similarity coefficient of ~ 0.497. Sub-cluster B_1_ contained four genotypes from Tripura, i.e. JRL1, CPG1, CPG5 and JRL2 and Sub-cluster B_2_ also comprised of 4 genotypes i.e. WNG2 and WNG3 from Meghalaya and SAIRANG1 and LENGTE1 from Mizoram. These results indicated that there is great intermixing between aonla genotypes which may be the result of cross-pollination. In addition, polymorphism in *P. emblica* may also be influenced by other geographical and environmental factors such as altitude and precipitation^29^. DARwin software was used for factorial analysis in which 4 genotypes from Tripura location were grouped likewise, 3 genotypes from Himachal Pradesh and 3 genotypes from Punjab. Similarly, the 5 commercial cultivars were found near to each other and 2 commercial cultivars (Banarsi and Krishna) grouped together as shown in dendrogram (Fig. 3). Moreover, the neighbor-joining cluster analysis (Fig. 4) from the results of DARwin software also showed different grouping of genotypes as earlier discussed in a dendrogram and factorial analysis like a grouping of different commercial cultivars, Tripura location's 4 genotypes, 3 genotypes of Himachal Pradesh, Banarsi and Krishna and also a group of 4 genotypes two from Meghalaya and rest of two from Mizoram location (Fig. 5). It can be emphasized from these findings that *P. emblica* exhibited high levels of polymorphism in the current study as it is widely distributed throughout the India and found in different habitats that have resulted in rich gene pool by everlasting adaptive evolution leading to high levels of genetic variation. Similarly, Liu et al.^30^ using 20 EST-SSR primers in 260 Chinese accessions of *P. emblica* established the presence of high levels of genetic diversity and low levels of genetic differentiation. Moreover, Rout et al.^31^ also carried out the cluster analysis using UPGMA of 12 species of *Phyllanthus* collected from different locations of India by using RAPD and ISSR markers that depicted high levels of genetic diversity.

Moreover, the corresponding sequences of the twelve (40%) EST-SSRs found to be polymorphic were BLAST against the GenBank nonredundant database using BLASTX and the top hits of all of them were found to be similar to different organisms, presented in Table 4. Out of these 12 EST-SSRs, 3 primers (PES-25, PES-28 and PES 30) code for enzymes that were involved in flavonoid biosynthesis pathway, 2 (PES-32 and PES-38) were involved in the synthesis of ascorbate and aldrate metabolism and one (PES-33) in calcium signaling pathway (Table 4). Similarly, Liu et al.^11^ reported twenty (38.5%) polymorphic EST-SSR markers. Out of 20 EST-SSRs, 7 shows top hits/similarity with transcription factor TCP7-like (*Jatropha curcas*), Sugar transporter ERD6-like 7 isoform X2 (*Jatropha curcas*), Hypothetical proteins from *Citrus clementina*, *Jatropha curcas* and *Sorghum bicolor*, 40S ribosomal protein S29, partial (*Zea mays)* and U-box domain-containing protein kinase family protein, putative (*Theobroma cacaos*) through BLASTX analysis.

**Table 4:** Sequence similarity details of EST-SSRs.

Sr. no	Primer	Unigene sequence similarity search	Enzyme name/function
1	PES-21	Transcription factor MYB, plant	Transcription factor
2	PES-25	*Manihot esculent* a chalcone synthase 2 (LOC110612862), mRNA (79.24%)	Chalcone synthase [EC:2.3.1.74]
3	PES-28	*Populuseuphratica* dihydroflavonol-4-reductase (LOC105113121), mRNA (76.82%)	Bifunctionaldihydroflavonol 4-reductase/flavanone 4-reductase [EC:1.1.1.219 1.1.1.234]
4	PES-30	*Camellia sinensis*chalcone–flavononeisomerase-like (LOC114317118), transcript variant X1, mRNA (73.61%)	Chalconeisomerase [EC:5.5.1.6]
5	PES-32	*Ricinus communis* l-galactono-1,4-lactone dehydrogenase, mitochondrial (LOC8286649), mRNA (82.06%)	l-galactono-1,4-lactone dehydrogenase [EC:1.3.2.3]
6	PES-33	*Populus trichocarpa* inositol monophosphatase 3 (LOC7458515), mRNA (83.76%)	Inositol-phosphate phosphatase/l-galactose 1-phosphate phosphatase [EC:3.1.3.25 3.1.3.93]
7	PES-35	*Vitis riparia* GDP-l-galactosephosphorylase 1-like (LOC117923648), transcript variant X4, mRNA (100%)	GDP-l-galactosephosphorylase [EC:2.7.7.69]
8	PES-40	*Ricinus communis*myb family transcription factor EFM (LOC8276198), mRNA (78.80%)	Mediate flower response
9	PES-42	*Populus alba* protein PHR1-LIKE 3-like (LOC118036633), transcript variant X1, mRNA (77.98%)	MYB family transcription factor
10	PES-44	*Ricinus communis* BIG SEEDS 1 (BS1) mRNA, complete cds (83.74%)	protein TIFY 4B like mRNA
11	PES-48	*Hevea brasiliensis* transcription factor bHLH48-like (LOC110670570), transcript variant X1, mRNA (78.18%)	Flower regulation
12	PES-49	*Juglans microcarpa* x *Juglans regia*bZIP transcription factor 60 (LOC121260009), mRNA (82.90%)	Transcription factor involved in the unfolded protein response UPR. Acts during endoplasmic reticulum stress (ER) by activating UPR target genes via direct binding to the UPR element (UPRE). Plays a role in plant immunity and abiotic stress responses

*ZIP* leucine zipper, *TIFY*-, *MYB* myeloblastosis, *mRNA* messenger ribonucleic acid, *HLH*-, *UPR* unfolded protein response, *GDP*-l-galactose phosphorylase.

## Conclusion

Exploring candidate genes involved in useful metabolic pathways is an important forward step towards better acceptability of the wild and commercial aonla to explore them in selecting superior genotypes. Thus, we developed novel EST-SSRs linked with secondary metabolisms which would be useful in investigating the population genetics, gene mining, population demographics, gene flow, and the genetic resource assessments of *P. emblica*. These findings would be contributing towards genetic structure of *P. emblica*, its evolutionary adaptations and genetic relationships among the closely related Phyllanthaceae species.

### Supplementary Information

Supplementary Figure 1.

Supplementary Figure 2.

Supplementary Figure 3.

Supplementary Figure 4.

Supplementary Table 1.

Supplementary Table 2.

Supplementary Table 3.

## Data Availability

The data sets generated during the current study are available in the NCBI database under the bioproject ID: PRJNA693681, BioSample: SAMN17394015 and SRA: SRR13512341 repository.
