# Supplementary figures and images for: De novo transcriptome profiling and development of novel secondary metabolites based genic SSRs in medicinal plant Phyllanthus emblica L. (Aonla)
Source: Sci Rep. 2023 Oct 12;13:17319. doi: 10.1038/s41598-023-44317-x (PMC10570353; doi:10.1038/s41598-023-44317-x)

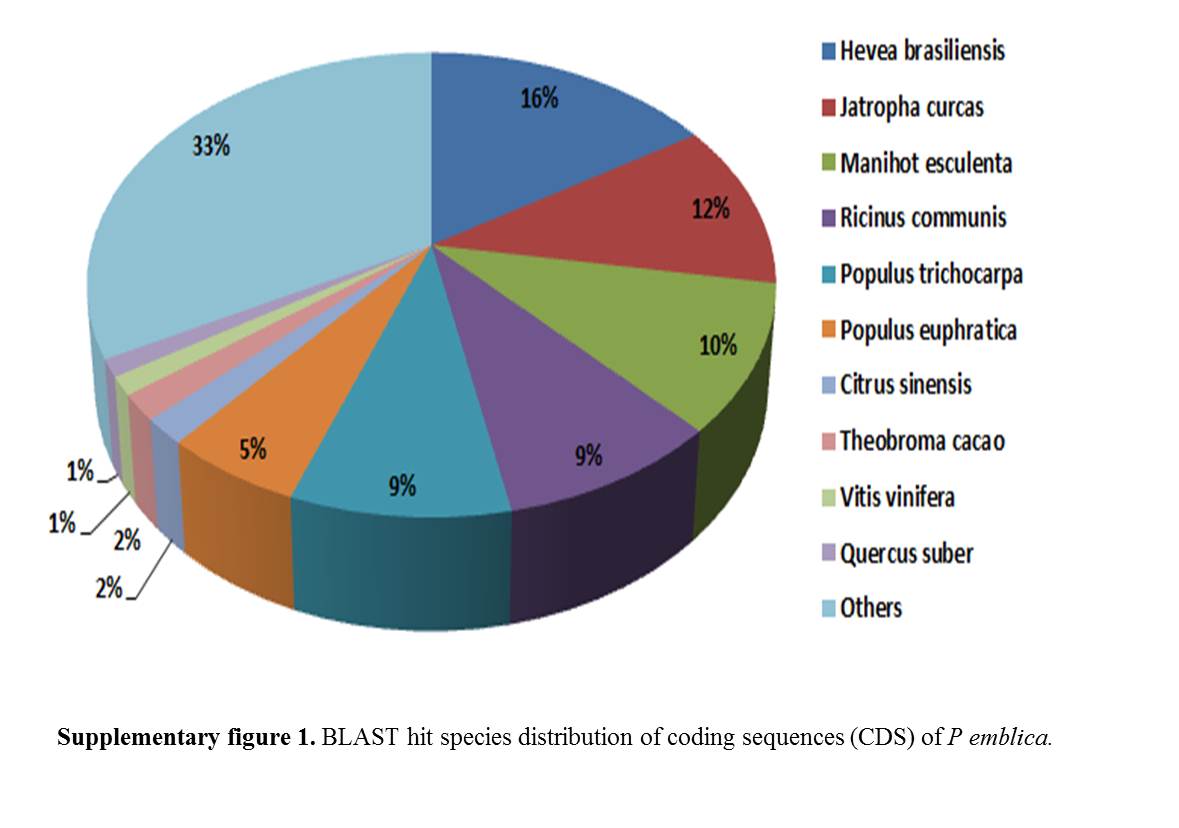

Supplement: Supplementary file 1 — Supplementary Figure 1. [file 41598_2023_44317_MOESM1_ESM.jpg]

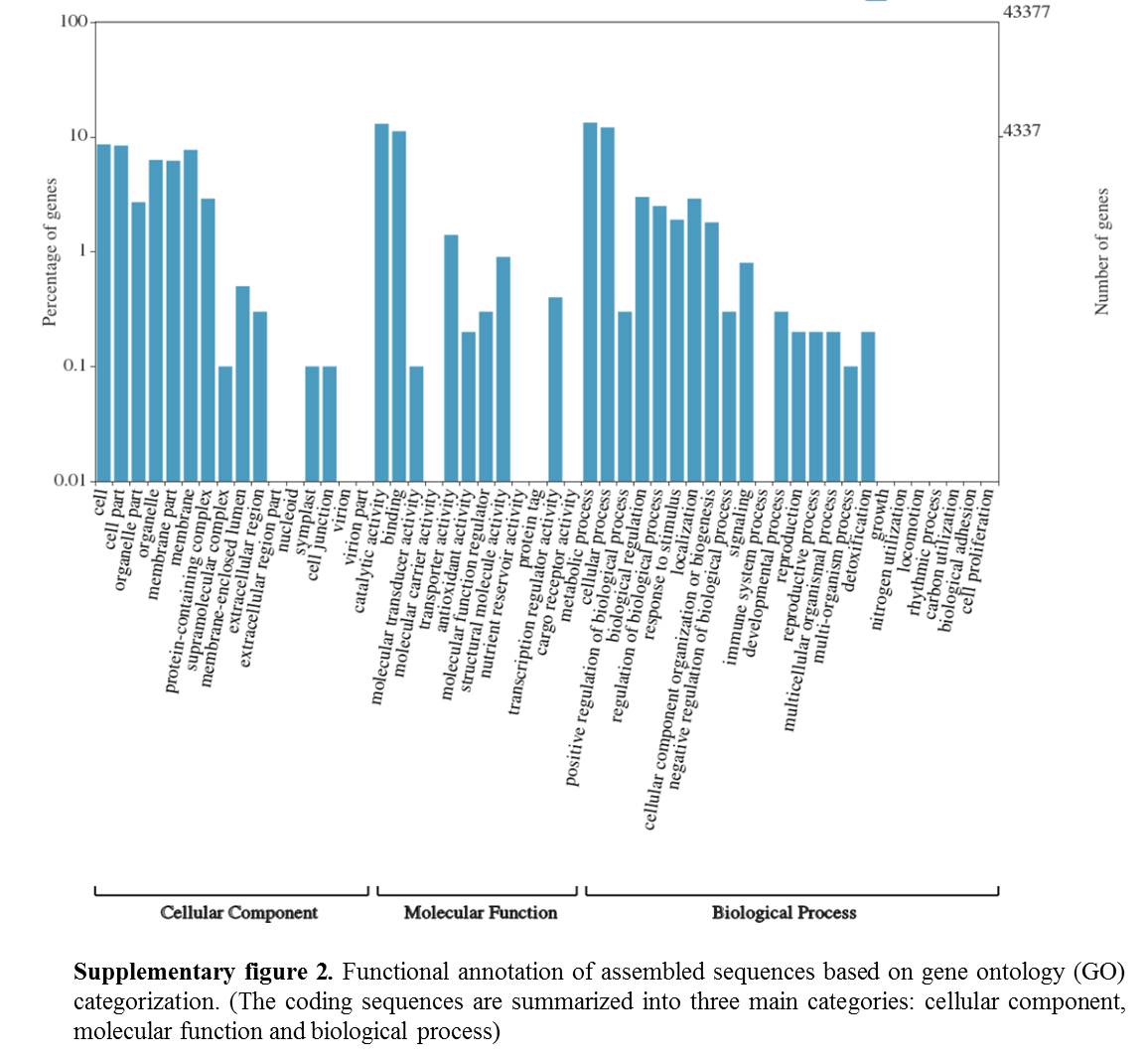

Supplement: Supplementary file 2 — Supplementary Figure 2. [file 41598_2023_44317_MOESM2_ESM.jpg]

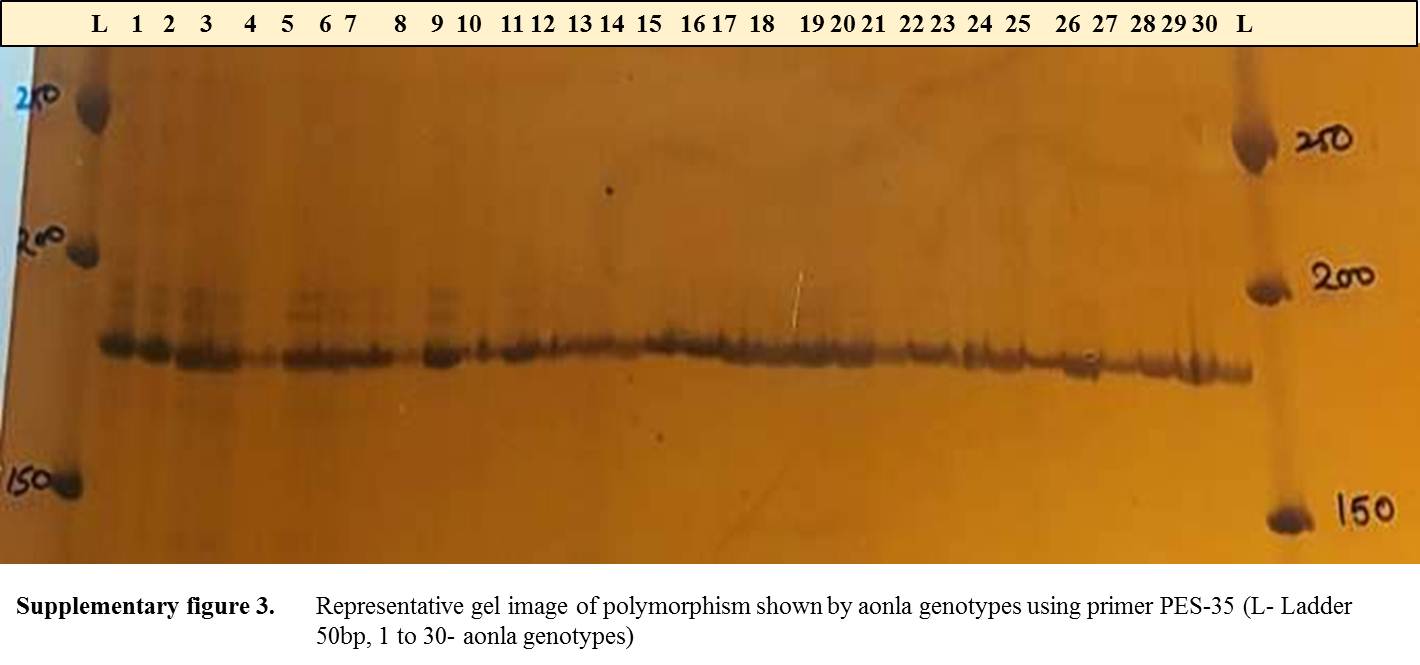

Supplement: Supplementary file 3 — Supplementary Figure 3. [file 41598_2023_44317_MOESM3_ESM.jpg]

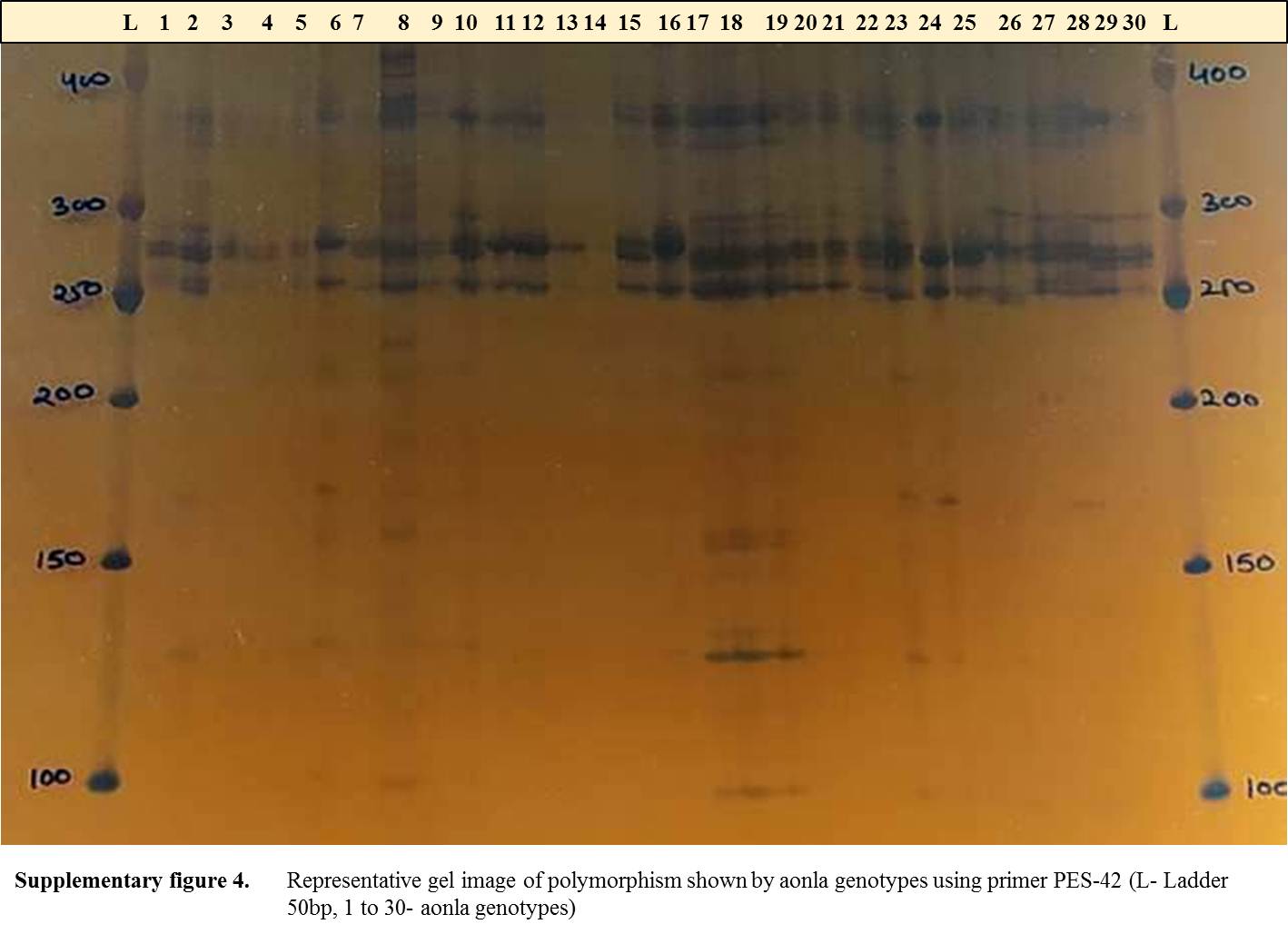

Supplement: Supplementary file 4 — Supplementary Figure 4. [file 41598_2023_44317_MOESM4_ESM.jpg]
