# Supplementary material for: De novo transcriptome profiling and development of novel secondary metabolites based genic SSRs in medicinal plant Phyllanthus emblica L. (Aonla)
Source: Sci Rep. 2023 Oct 12;13:17319. doi: 10.1038/s41598-023-44317-x (PMC10570353; doi:10.1038/s41598-023-44317-x)
Supplement: Supplementary file 5 — Supplementary Table 1. [file 41598_2023_44317_MOESM5_ESM.docx]

| **Sr. No.** | **Accessions** | **Type** | **Origin/ Source** |
| --- | --- | --- | --- |
|  | NA-6 | Seedling selection of Chakaiya | Narendra Dev University of Agriculture and Technology (NDUAT), Kumarganj, Faizabad, Uttar Pradesh, India |
|  | NA-7 | Clone of Francis | Narendra Dev University of Agriculture and Technology (NDUAT), Kumarganj, Faizabad, Uttar Pradesh, India |
|  | NA-10 | Chance seedling of Banarasi | Narendra Dev University of Agriculture and Technology (NDUAT), Kumarganj, Faizabad, Uttar Pradesh, India |
|  | L-52 (Laxmi-52) | Superior Chance seedling of Francis | Originated from Pratapgarh, Uttar Pradesh, India |
|  | CHAKIYA | Natural seedling selection | Varanasi district of Uttar Pradesh, India |
|  | KANCHAN (NA-5) | Seedling selection of Chakaiya | Narendra Dev University of Agriculture and Technology (NDUAT), Kumarganj, Faizabad, Uttar Pradesh, India |
|  | KRISHNA (NA-4) | Chance seedling of Banarasi | Originated from Pratapgarh, Uttar Pradesh, India |
|  | BANARSI | Natural seedling selection | Varanasi district of Uttar Pradesh, India |
|  | HATHIJHOOL(FRANCIS) | Natural seedling selection | Originated from Pratapgarh, Uttar Pradesh, India |
|  | PUN1 (PUN) | Wild | Punjab Agricultural University, Ludhiana, Punjab, India |
|  | PUN2 (PUN) | Wild | Punjab Agricultural University, Ludhiana, Punjab, India |
|  | PUN3 (PUN) | Wild | Punjab Agricultural University, Ludhiana, Punjab, India |
|  | HPK2(HP) | Wild | Kangra, Himachal Pradesh, India |
|  | HPK3(HP) | Wild | Kangra, Himachal Pradesh, India |
|  | HPK4(HP) | Wild | Kangra, Himachal Pradesh, India |
|  | HPH3(HP) | Wild | Hamirpur, Himachal Pradesh, India |
|  | HPH4(HP) | Wild | Hamirpur, Himachal Pradesh, India |
|  | HPH5(HP) | Wild | Hamirpur, Himachal Pradesh, India |
|  | TPN1 | Wild | Tepania Eco Park, Tripura, India |
|  | TPN7 | Wild | Tepania Eco Park, Tripura, India |
|  | JRL1 | Wild | Jarulcherra, Tripura, India |
|  | CPG1 | Wild | Champaknagar, Tripura, India |
|  | CPG5 | Wild | Champaknagar, Tripura, India |
|  | JRL2 | Wild | Jarulcherra, Tripura, India |
|  | UMR 1 | Wild | Umroi, Meghalaya, India |
|  | UMR 2 | Wild | Umroi, Meghalaya, India |
|  | WNG 2 | Wild | Williamnagar, Meghalaya, India |
|  | WNG 3 | Wild | Williamnagar, Meghalaya, India |
|  | SAIRANG 1 | Wild | Sairang, Mizoram, India |
|  | LENGTE | Wild | Lengte, Mizoram, India |

**Supplementary Table 1.** Details of *P. emblica* accessions used in the study along with their type and source information
