# Supplementary material for: De novo transcriptome profiling and development of novel secondary metabolites based genic SSRs in medicinal plant Phyllanthus emblica L. (Aonla)
Source: Sci Rep. 2023 Oct 12;13:17319. doi: 10.1038/s41598-023-44317-x (PMC10570353; doi:10.1038/s41598-023-44317-x)
Supplement: Supplementary file 6 — Supplementary Table 2. [file 41598_2023_44317_MOESM6_ESM.docx]

| **Sr. No** | **Coding Seq. No.** | **Gene** | **EC number** |
| --- | --- | --- | --- |
|  | CDS_13956 | chalcone synthase | [EC:2.3.1.74] |
|  | CDS_13959 | chalcone synthase | [EC:2.3.1.74] |
|  | CDS_37928 | chalcone synthase | [EC:2.3.1.74] |
|  | CDS_37929 | chalcone synthase | [EC:2.3.1.74] |
|  | CDS_15163 | chalcone isomerase | [EC:5.5.1.6] |
|  | CDS_21393 | chalcone isomerase | [EC:5.5.1.6] |
|  | CDS_21394 | chalcone isomerase | [EC:5.5.1.6] |
|  | CDS_21395 | chalcone isomerase | [EC:5.5.1.6] |
|  | CDS_11782 | naringenin 3-dioxygenase | [EC:1.14.11.9] |
|  | CDS_11784 | naringenin 3-dioxygenase | [EC:1.14.11.9] |
|  | CDS_12925 | flavonol synthase | [EC:1.14.20.6] |
|  | CDS_5289 | trans-cinnamate 4-monooxygenase | [EC:1.14.14.91] |
|  | CDS_30207 | trans-cinnamate 4-monooxygenase | [EC:1.14.14.91] |
|  | CDS_30208 | trans-cinnamate 4-monooxygenase | [EC:1.14.14.91] |
|  | CDS_20065 | flavonoid 3'-monooxygenase | [EC:1.14.14.82] |
|  | CDS_17008 | bifunctional dihydroflavonol 4-reductase/ flavanone 4-reductase | [EC:1.1.1.219 1.1.1.234] |
|  | CDS_34204 | bifunctional dihydroflavonol 4-reductase/  flavanone 4-reductase | [EC:1.1.1.219 1.1.1.234] |
|  | CDS_34205 | bifunctional dihydroflavonol 4-reductase/  flavanone 4-reductase | [EC:1.1.1.219 1.1.1.234] |
|  | CDS_34206 | bifunctional dihydroflavonol 4-reductase/  flavanone 4-reductase | [EC:1.1.1.219 1.1.1.234] |
|  | CDS_22638 | flavonoid 3',5'-hydroxylase | [EC:1.14.14.81] |
|  | CDS_22639 | flavonoid 3',5'-hydroxylase | [EC:1.14.14.81] |
|  | CDS_19905 | anthocyanidin synthase | [EC:1.14.20.4] |
|  | CDS_19908 | anthocyanidin synthase | [EC:1.14.20.4] |
|  | CDS_40216 | anthocyanidin reductase | [EC:1.3.1.77] |
|  | CDS_34864 | leucoanthocyanidin reductase | [EC:1.17.1.3] |
|  | CDS_5127 | flavanone 7-O-glucoside 2''-O-beta- L rhamnosyltransferase | [EC:2.4.1.236] |
|  | CDS_11566 | phlorizin synthase | [EC:2.4.1.357] |
|  | CDS_11568 | phlorizin synthase | [EC:2.4.1.357] |
|  | CDS_11569 | phlorizin synthase | [EC:2.4.1.357] |
|  | CDS_3323 | shikimate O-hydroxycinnamoyltransferase | [EC:2.3.1.133] |
|  | CDS_8122 | shikimate O-hydroxycinnamoyltransferase | [EC:2.3.1.133] |
|  | CDS_24191 | shikimate O-hydroxycinnamoyltransferase | [EC:2.3.1.133] |
|  | CDS_32256 | shikimate O-hydroxycinnamoyltransferase | [EC:2.3.1.133] |
|  | CDS_32258 | shikimate O-hydroxycinnamoyltransferase | [EC:2.3.1.133] |
|  | CDS_32260 | shikimate O-hydroxycinnamoyltransferase | [EC:2.3.1.133] |
|  | CDS_32261 | shikimate O-hydroxycinnamoyltransferase | [EC:2.3.1.133] |
|  | CDS_3323 | shikimate O-hydroxycinnamoyltransferase | [EC:2.3.1.133] |
|  | CDS_8122 | shikimate O-hydroxycinnamoyltransferase | [EC:2.3.1.133] |
|  | CDS_24191 | shikimate O-hydroxycinnamoyltransferase | [EC:2.3.1.133] |
|  | CDS_34418 | shikimate O-hydroxycinnamoyltransferase | [EC:2.3.1.133] |
|  | CDS_34420 | shikimate O-hydroxycinnamoyltransferase | [EC:2.3.1.133] |
|  | CDS_30981 | 5-O-(4-coumaroyl)-D-quinate 3'-monooxygenase | [EC:1.14.14.96] |
|  | CDS_26389 | caffeoyl-CoA O-methyltransferase | [EC:2.1.1.104] |
|  | CDS_26391 | caffeoyl-CoA O-methyltransferase | [EC:2.1.1.104] |
|  | CDS_26392 | caffeoyl-CoA O-methyltransferase | [EC:2.1.1.104] |
|  | CDS_29584 | caffeoyl-CoA O-methyltransferase | [EC:2.1.1.104] |
|  | CDS_29585 | caffeoyl-CoA O-methyltransferase | [EC:2.1.1.104] |
|  | CDS_29586 | caffeoyl-CoA O-methyltransferase | [EC:2.1.1.104] |
|  | CDS_29588 | caffeoyl-CoA O-methyltransferase | [EC:2.1.1.104] |
|  | CDS_5127 | flavanone 7-O-glucoside 2''-O-beta-L-rhamnosyltransferase | [EC:2.4.1.236] |
|  | CDS_22638 | flavonoid 3',5'-hydroxylase | [EC:1.14.14.81] |
|  | CDS_22639 | flavonoid 3',5'-hydroxylase | [EC:1.14.14.81] |
|  | CDS_20065 | flavonoid 3'-monooxygenase | [EC:1.14.14.82] |
|  | CDS_5510 | flavonol 3-O-glucosyltransferase | [EC:2.4.1.91] |
|  | CDS_37740 | flavonol-3-O-glucoside/galactoside glucosyltransferase | [EC:2.4.1.239 2.4.1.-] |
|  | CDS_37741 | flavonol-3-O-glucoside/galactoside glucosyltransferase | [EC:2.4.1.239 2.4.1.-] |
|  | CDS_37743 | flavonol-3-O-glucoside/galactoside glucosyltransferase | [EC:2.4.1.239 2.4.1.-] |
|  | CDS_4489 | flavonol-3-O-glucoside L-rhamnosyltransferase | [EC:2.4.1.159] |
|  | CDS_2892 | isoflavone/4'-methoxyisoflavone 2'-hydroxylase | [EC:1.14.14.90 1.14.14.89] |
|  | CDS_7635 | isoflavone/4'-methoxyisoflavone 2'-hydroxylase | [EC:1.14.14.90 1.14.14.89] |
|  | CDS_31279 | isoflavone/4'-methoxyisoflavone 2'-hydroxylase | [EC:1.14.14.90 1.14.14.89] |
|  | CDS_31281 | isoflavone/4'-methoxyisoflavone 2'-hydroxylase | [EC:1.14.14.90 1.14.14.89] |

**Supplementary Table 2.** Genes associated with the production of flavonoids identified in *P. emblica* transcriptome
