# Supplementary material for: De novo transcriptome profiling and development of novel secondary metabolites based genic SSRs in medicinal plant Phyllanthus emblica L. (Aonla)
Source: Sci Rep. 2023 Oct 12;13:17319. doi: 10.1038/s41598-023-44317-x (PMC10570353; doi:10.1038/s41598-023-44317-x)
Supplement: Supplementary file 7 — Supplementary Table 3. [file 41598_2023_44317_MOESM7_ESM.docx]

| **Repeat Motif** | **Repeat Number** | | | | | | | | | | **Percentage (%)** |
| --- | --- | --- | --- | --- | --- | --- | --- | --- | --- | --- | --- |
|  | **4** | **5** | **6** | **7** | **8** | **9** | **10** | **11** | **>11** | **Total** |  |
| Dinucleotide |  |  |  |  |  |  | 263 | 228 | 485 | 976 | 13.05 |
| Trinucleotide | 3883 | 942 | 428 | 226 | 125 | 30 | 23 | 13 | 32 | 5702 | 76.27 |
| Tetranucleotide |  | 84 | 20 | 3 |  | 1 |  | 1 | 1 | 110 | 1.48 |
| Pentanucleotide |  | 41 | 5 | 4 |  |  |  |  |  | 50 | 0.67 |
| Hexanucleotide |  | 6 |  |  |  |  |  |  |  | 6 | 0.08 |
| Composite type |  |  |  |  |  |  |  |  |  | 633 | 8.46 |
| Total | 3883 | 1073 | 453 | 233 | 125 | 31 | 286 | 242 | 518 | 7477 |  |
| Percentage (%) | 51.94 | 14.36 | 6.06 | 3.12 | 1.68 | 0.42 | 3.83 | 3.24 | 6.93 |  |  |

**Supplementary Table 3.** Per cent distribution of nucleotide repeats in *P. emblica.* transcriptome
